# Adaptation to Different Human Populations by HIV-1 Revealed by Codon-Based Analyses

**DOI:** 10.1371/journal.pcbi.0020062

**Published:** 2006-06-23

**Authors:** Sergei L Kosakovsky Pond, Simon D. W Frost, Zehava Grossman, Michael B Gravenor, Douglas D Richman, Andrew J. Leigh Brown

**Affiliations:** 1 Department of Pathology, University of California San Diego, La Jolla, California, United States of America; 2 National HIV Reference Lab, Public Health Laboratory, Ministry of Health, Tel Hashomer, Israel; 3 School of Medicine, University of Swansea, Swansea, Wales, United Kingdom; 4 VA San Diego Health Care System, San Diego, California, United States of America; 5 Institute for Evolutionary Biology, School of Biological Sciences, University of Edinburgh, Edinburgh, Scotland, United Kingdom; The Pennsylvania State University, United States of America

## Abstract

Several codon-based methods are available for detecting adaptive evolution in protein-coding sequences, but to date none specifically identify sites that are selected differentially in two populations, although such comparisons between populations have been historically useful in identifying the action of natural selection. We have developed two fixed effects maximum likelihood methods: one for identifying codon positions showing selection patterns that persist in a population and another for detecting whether selection is operating differentially on individual codons of a gene sampled from two different populations. Applying these methods to two HIV populations infecting genetically distinct human hosts, we have found that few of the positively selected amino acid sites persist in the population; the other changes are detected only at the tips of the phylogenetic tree and appear deleterious in the long term. Additionally, we have identified seven amino acid sites in protease and reverse transcriptase that are selected differentially in the two samples, demonstrating specific population-level adaptation of HIV to human populations.

## Synopsis

Despite the efforts devoted to surveying HIV genetic diversity and the development of an effective vaccine, there is still no consensus on the extent to which the former prejudices the latter. Experimental studies show that escape from cell-mediated immunity is selected for in HIV and SIV, and sometimes very quickly. Conversely, escape mutants may be selected against at transmission, so how much does this selection within individuals influence the genotype of the circulating HIV population overall? Kosakovsky Pond, Leigh Brown, and colleagues have developed a new statistical approach to address this question. Using sequences from the globally most abundant HIV subtype (subtype C), the authors directly compared virus of the same subtype infecting genetically different human populations. They show at least half of the amino acid sites selected within individuals are not selected at a population level, and they identify six amino acid sites in the RT gene that are selected differentially between populations. We can now partition molecular adaptation between individual and population components for whatever genes may be included in candidate vaccines, in the target populations themselves. The methods are also important beyond the HIV world, where analogous issues arise in the more general question of the evolution of virulence in pathogens.

## Introduction

Differences in allele frequencies in different populations were used as evidence of natural selection in some classic studies [1–3]. Since the first identification of positive selection within protein sequences [4,5], however, estimation of the relative frequency of synonymous and non-synonymous nucleotide substitution has become a standard tool in molecular evolutionary studies. The power of these analyses to detect selection was substantially increased through the development of codon-based likelihood models that allow selection to vary across sites [6]. We have developed a method, within a maximum likelihood framework, that combines these two approaches to yield novel insights into adaptive evolutionary differences between populations. We have applied this method to investigate the hypothesis that HIV has adapted specifically to the distinct human host population.

The immune system is widely recognized as one of the factors that exert a selective effect on pathogen populations. Mutations that allow HIV-1 strains to escape MHC-restricted CTL killing arise in both acute and chronic infection [7–11], but in the absence of an immune response they can reduce fitness [12], and can be selected against on transmission [13,14]. Recently, from correlations of variability and CTL epitopes, it was suggested that adaptation to human CTL responses had led to genetic adaptations in the HIV genome [15]. Both individual- and population-based studies have found overwhelming evidence of positive selection in HIV [6,16–22] but have been unable to discriminate between possible mechanisms. Generally speaking, phylogenetic studies that rely on within-population sequence polymorphism to identify non-neutral evolution may not be able to detect selective sweeps, expressed by substitutions localized to a single branch of the phylogeny of serially sampled sequences; selected alleles driven to fixation in all sequences of the sample, resulting in within-population monomorphic sites; or the direction of selection, for instance acquisition of immune escape mutations versus reversion in the absence of selective forces [23].

We have developed a suite of fixed effects likelihood-based approaches [22,24] which we have used here to discriminate substitutions occurring on internal branches from those occurring at the tips of the tree, and to estimate whether selective pressure at a given site is different between two populations of HIV-infected individuals. This allows one to distinguish sites that are positively selected because they are associated with adaptation to the individual host from those associated with adaptation to the population.

## Results

We analyzed the sequences of the protease (PR) and reverse transcriptase (RT) coding regions of HIV clade C from each of 74 individuals from KwaZulu Natal [25] and Zambia (ZA sample), and from 63 Ethiopian Falasha immigrants arriving in Israel between 1998 and 2003 [26] (ET sample). These sequences were obtained by population-based sequencing from viral RNA PCR-amplified direct from the blood plasma of the infected individual: each sequence thus reflects the predominant nucleotides in the plasma viral population of that individual at that time. Within-host recombination will not affect a phylogeny based on these sequences. Our maximum likelihood calculations treated ambiguities as partially missing data. Amino acid positions 4 through 99 of PR and 41 through 240 of RT were analyzed. The absence of first 40 positions in RT and three positions in PR is an artifact of the sequencing procedure used for generating some sequences in the ET sample. The alignments used in this study did not contain any insertions or deletions, which is common for HIV *pol* alignments. We screened all sequences for mutations known to confer strong drug resistance and found that none of the sequences included in the study had such mutations [27–29]. Each sample formed a separate clade (Figure S1) in the maximum likelihood tree built from PR and RT jointly (reconstructed using the GTR+G+I model with the PhyML package [30]). This clustering was confirmed by Bayesian phylogenetic reconstruction, performed under the same model using the MrBayes software package [31]. 10^6^ MCMC samples, thinned by a factor of 100, were generated, and the first 25% of the samples were discarded as burn-in. Sequence alignments and maximum likelihood trees for each sample can be downloaded (in NEXUS format) from http://www.hyphy.org/pubs/DS/sequences.tgz.

Phylogenetic methods can perform poorly if viral sequences have experienced sufficient recombination to result in discordant phylogenetic signal in different parts of the alignment [32,33]. We carried out a simple procedure, similar to the ideas in [34] and [35], to screen for evidence of recombination. Given an alignment, we split the data into two contiguous fragments, reconstructed neighbor joining trees [36] for each segment, fitted the trees using maximum likelihood, and investigated whether having two trees improves the small sample AIC score [37] over the model with one tree for the entire alignment. Additionally, we verified phylogenetic incongruence using the Shimodaira–Hasegawa [38] test. This test was carried out for all possible placements of the breakpoint. For the four alignments in this study, no two-tree model fitted the data significantly better than the single-tree model and yielded a significant SH test result. While this screening procedure does not rule out the presence of recombinant sequences in our alignments, it suggests that the impact of recombination is insufficient to cause detectably discordant phylogenies, and to heavily bias the inference procedure. As an additional check, we carried out the RDP test [39], which also failed to detect any recombination events in any of the four alignments.

Gene by gene maximum likelihood codon rate analyses revealed strong rate heterogeneity of both synonymous and nonsynonymous substitution rates in all four samples (Table 1). Consequently, it is imperative that site-to-site variation in synonymous rates be accounted for, lest the tests for selection suffer high rates of false positives [22,40]. Using three independent maximum likelihood methods for detecting selection at an individual site [22], we identified four sites in PR and seven sites in RT subject to significant positive selection, as detected by the consensus of the methods. Codons 12, 19, and 63 in PR were positively selected in both populations, as were codons 48, 166, 173, and 207 in RT. Other sites gave significant results using only one test, or were positively selected in only one population (Table 2).

**Table 1:**
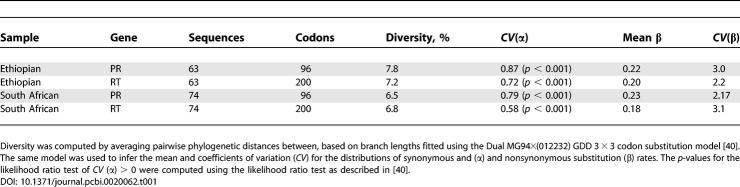
Sequences Analyzed in This Study

**Table 2:**
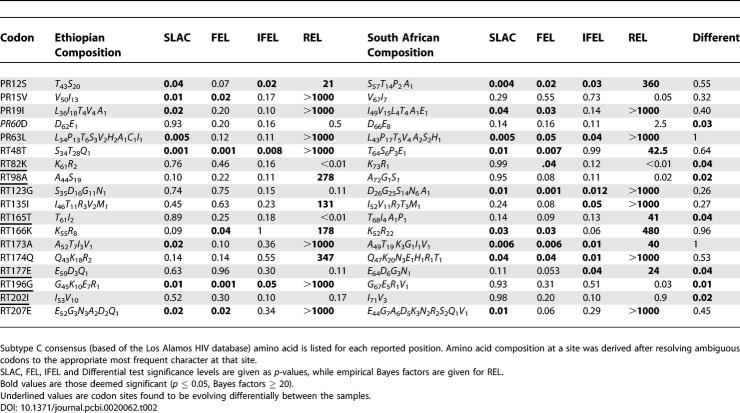
Sites found to be under positive and/or differential selection.

### Individual versus Population-Level Adaption in HIV

**Figure 1:**
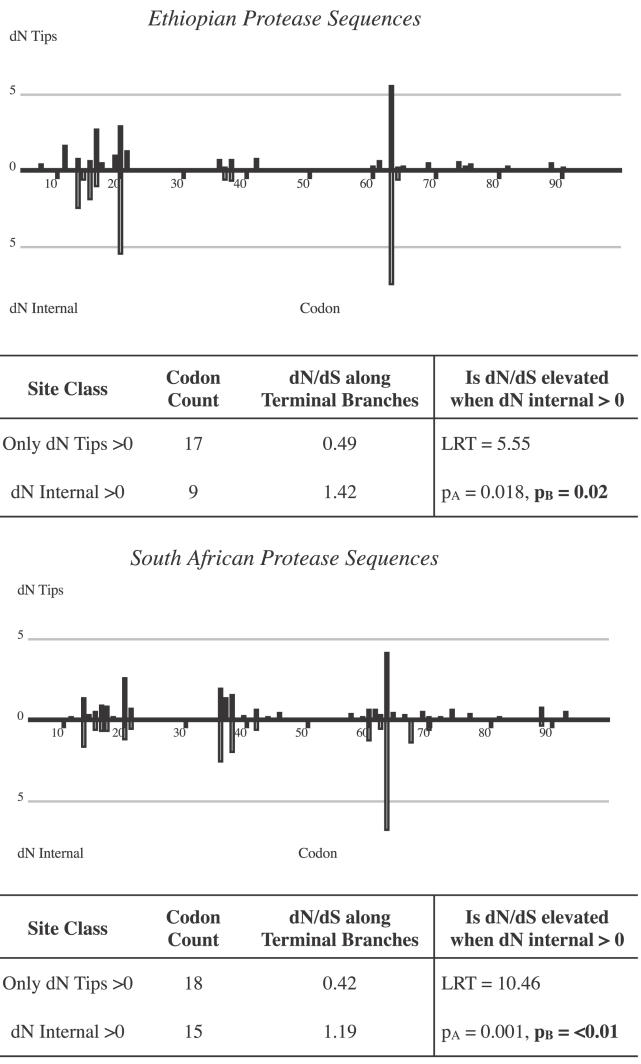
Patterns of Population and Individual Level Nonsynonymous Evolution in the Two Protease Samples MLEs of nonsynonymous substitution rates along internal (dN Internal) and terminal (dN Tips) branches were derived using the IFEL method. *dN/dS* estimates and *p*-values in the tables were obtained with the Population Level Adaptation test. *p_A_* and *p_B_* and denote, respectively, the asymptotic and parametric bootstrap significance levels for the likelihood ratio test.

**Figure 2:**
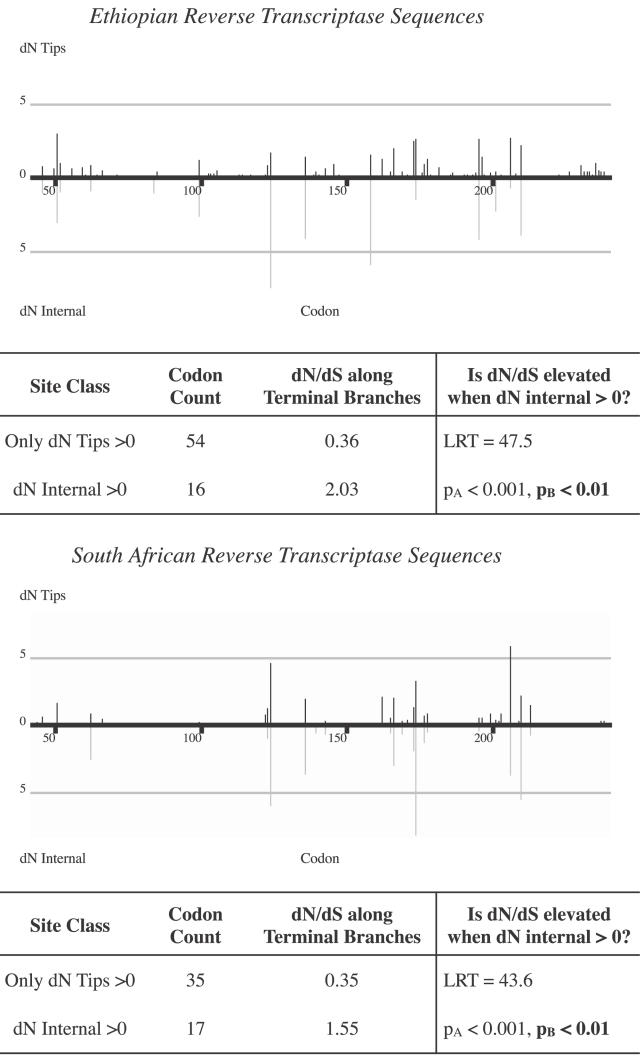
Patterns of Population and Individual Level Nonsynonymous Evolution in the Two RT Samples MLEs of nonsynonymous substitution rates along internal (*dN* Internal) and terminal (*dN* Tips) branches were derived using the IFEL method. *dN/dS* estimates and *p*-values in the tables were obtained with the Population Level Adaptation test. *p_A_* and *p_B_* denote, respectively, the asymptotic and parametric bootstrap significance levels for the likelihood ratio test.

Sharp et al. [41] previously estimated *dN/dS* ratios for all branches in a phylogeny of HIV-1 and SIVcpz *env* sequences and correlated that quantity with the “depth” of the branch in the tree to deduce that *dN/dS* across the entire sequence was smaller for branches that were far from the tips of the tree. Holmes [42] performed alignment-wide comparisons of dengue virus sequences sampled from individual hosts and from populations of infected hosts and found that average selective pressures were substantially more purifying in between-host samples. We have extended one of the methods used to detect positive selection (fixed effects likelihood) to permit the estimation of the ratio of nonsynonymous (*β* or *dN*) and synonymous substitution rates (*α* or *dS*) separately in internal and terminal branches of the tree connecting these sequences. This has revealed that many recent nonsynonymous substitutions, i.e., those in the terminal branches of the tree, were not represented on internal branches. For both the ZA and ET populations, there are more codons in both PR and RT with only recent nonsynonymous substitutions than there are codons with substitutions on internal branches (Figures 1 and 2). The difference was particularly striking in RT where the ratio was 35:17 in the ZA sample and 54:16 in the ET sample. This disparity alone may be statistically insignificant because the cumulative length of internal branches in the tree is smaller than that of terminal branches (Figure S1). However, at those codons where internal substitutions are seen, the strength of selection (measured by the *dN*/*dS* ratio) along terminal branches is in all cases higher (Figures 1 and 2): in all four comparisons this difference was significant based on the likelihood ratio test (*p* < 0.05 using parametric bootstrap to guard against the effect of small sample sizes).

At the level of individual sites, three sites were positively selected (*p* ≤ 0.05) along internal branches in the ET sample and seven in the ZA sample. Simulation results (see Materials and Methods) suggest that the test is conservative for model parameters chosen to resemble those likely to have generated our samples, and is capable of reliably detecting sites that are subject to strong selection. Positive predictive value (PPV) of the test was calculated at 98.8%, hence it is unlikely that detected sites are false positives. In particular, a high PPV estimate strongly suggests that site-wise testing procedures in this context do not require a correction for multiple testing.

Positively selected nonsynonymous substitutions on internal branches (persistent substitutions) must of necessity be adaptive at both individual host and population level. As we have analysed consensus sequences of the within-individual populations, the substitutions must have reached a high frequency in the infected individuals, but are transient at the population level, suggesting their removal by purifying selection. Based on the elevated rate of adaptation within individuals detected at codons subject to population-level selection, relative to the codons where only recent substitutions have been inferred, we conclude that recent substitutions are, on average, maladaptive at the level of the human population. We note that when longitudinal data are not available, comparative phylogenetic methods may be unable to detect directional selection if the population had undergone a selective sweep. Population level adaptation inferred for our samples could also be due to transient directional selection, or diversifying selection maintained by acquisition and transmission of escape mutants and reversion to wild type. However, because time scales of transmission and reversion processes are not known for this sample, a single mechanism cannot be distinguished.

### Differential Adaptive Evolution in Different Populations

Human major histocompatibility complex (MHC) alleles are remarkably old, and some have been maintained since the human–chimpanzee divergence [43]. Thus, adaptation to human MHC alleles may not only reflect adaptation since the zoonotic transfer of HIV from chimpanzee to humans, but also include the prior history in the chimpanzee population. However, differential adaptation to different human populations could only be due to a species-specific process. Although many comparisons between HIV sequences from different host populations are confounded by a substantial phylogenetic difference between the viral populations, HIV-1 M group clade C has infected a number of ethnically distinct populations. We compared the sequence dataset from southern Africa with another subtype C dataset sampled from Ethiopian Falasha immigrants arriving in Israel between 1998 and 2003 [26]. An earlier study has shown that the Falasha (Amharic) share most genetic markers with other Ethiopian groups [44], and Ethiopian populations have quite distinct allele frequency spectra at HLA loci [45–47]. This comparison allows us to test the explicit hypothesis that passage through different human populations has led to adaptive divergence in the virus genome.

As transient substitutions would not contribute to interpopulation adaptive divergence, only internal branches were tested for population-specific positive selection. A novel maximum likelihood test for differential selection (see Materials and Methods) permits the direct comparison of selection pressures on individual amino acid sites between populations. The test takes into account nucleotide substitution biases and weights over all possible ancestral codons, while avoiding assumptions regarding the distribution of dN and dS across sites. With this test we identified one codon in PR (60) and six codons in RT (82, 98, 165, 177, 196, and 202) as selected differentially in the two populations, at *p* ≤ 0.05. Thus there is evidence for differential selection between these two populations at seven codons out of 296 compared in RT and PR. Based on the high (98.2%) PPV of the test achieved on simulated data (see Materials and Methods) and the overall low power of the test for relatively small sample sizes, we conclude that these seven codons are unlikely to be false positive results, and that they probably constitute only a portion of codons which evolve differentially between the samples.

**Figure 3:**
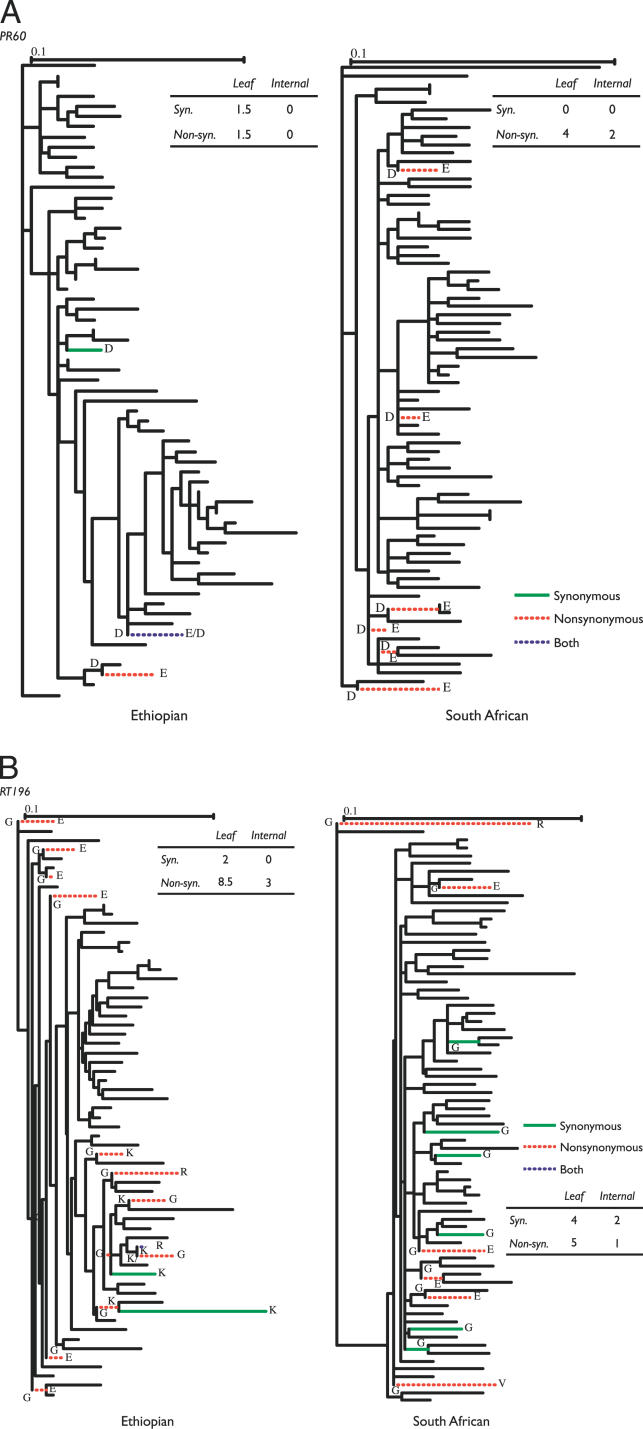
Inferred Evolutionary History at Two Codons in *pol* (A) Codon 60 in protease. The trees were rooted using subtype B reference sequences from the Los Alamos HIV database, and ancestral states were inferred using maximum likelihood under the MG94×012232 Dual GDD 3 × 3 [40] model of codon evolution. (B) Codon 196 in RT. The trees were rooted using subtype B reference sequences from the Los Alamos HIV database, and ancestral states were inferred using maximum likelihood under the MG94×012232 Dual GDD 3 × 3 [40] model of codon evolution.

Figure 3A and 3B (and Figures S2–S6) show a codon-based maximum likelihood reconstruction of evolutionary history at these codon positions. We note that at many codons, evolution in both populations involves the same residues, but drastically different patterns of substitutions throughout the tree, with one population showing synonymous and nonsynonymous evolution along terminal branches only, while the other displays nonsynonymous substitutions along internal branches as well as ongoing evolution at the tips (e.g., Figure 3B).

We also note that some sites (e.g., PR12 and RT48 in Table 2) show evidence of selection in both samples, but sequences appear to be driven towards different residues in each sample. Our method does not distinguish such sites as differentially selected, because they are subject to similar selective pressures in both samples, regardless of which residue appears to be selected for.

## Discussion

The MHC-restricted host immune response represents a continuous selective force on pathogens whose effect is dependent on the pathogen genotype. In the case of HIV, viral escape mutations can arise soon after infection and can be transmitted onward, when their fate will depend on the MHC genotype of the new host [13]. In the absence of an active CTL response, due to MHC discordance, such escape mutations can be lost relatively quickly, implying that a second, antagonistic, selective force acts on the same genetic variant, possibly replication rate [48,49]. The extent to which the HIV and other viral genomes are shaped by the human immune response will therefore depend on the balance between these two effects. Only those mutations that either do not incur a significant cost in replicative efficiency, or have such a low probability of being recognized in the human population, would persist at the population level, and such population-level adaptation would be observed on internal branches of a phylogenetic tree of viral sequences.

Analyzing viral *pol* gene sequences from two populations infected with HIV subtype C, we have found many codons with amino acid substitutions only at the tips. At these sites variation is much lower than at those with both internal and tip substitutions, suggesting long-term purifying selection has removed many recent substitutions that may have arisen as adaptations to individual hosts. This suggests there are substantial long-term constraints on the extent to which the genome of HIV can be modified by human MHC-restricted immune responses. However, at seven codons there is evidence that substitutions on internal branches are selected differentially between the two human populations studied, confirming that these constraints are sequence context–specific. As the density of CTL epitopes in *pol* is low relative to that of other genes such as *gag, tat,* and *nef* [50,51], the level of population adaptation in other genes could well be even higher.

Our approach looks for differences in evolutionary forces exerted deep in the phylogenetic trees, which are not always readily manifested in the amino-acid composition at a given site, or raw numbers of inferred synonymous or nonsynonymous substitutions. This approach can augment simpler but less sensitive methods [15,23], which rely on observed amino-acid composition of a site, or on detecting mutations toward (reversion) or away from (escape) a reference sequence (e.g., subtype consensus), thought to represent a variant with higher fitness in absence of selection.

Our methodology offers an alternative and more general approach to the “branch-site” class methods [52], which attempt to identify site-by-site positive selection along a single branch using a random effects approach and empirical Bayes inference. For example, Travers et al. [21] used such methods to locate sites under selection along predefined branches in a phylogeny of HIV-1 sequences from different clades. Our approaches are able to test selection operating along a set of tree branches, without assuming an a priori, perhaps unnecessarily restrictive, parametric form for all possible selection regimes. For instance, Bielawski and Yang [53] assumed that there are at most three modes of selection, with fixed selection strength at every site in a given mode. In contrast, by adopting a fixed effects phylogenetic likelihood framework [22] and inferring various selection regimes directly at every site, we can sidestep the problems inherent in model mis-specification in the context of branch-site models [54,55] and uncertainties associated with phylogenetic empirical Bayes inference in general.

In addition, we have developed a novel test to identify differential adaptation in different populations. This test is particularly suitable for exploring adaptation of parasites to genetically different host populations, and it allowed us to identify a subset of amino acid sites in PR and RT coding regions of HIV that were differentially selected in two human populations. Previous studies [56] have drawn upon observed correlations between location of sites subject to selection to hypothesize concordant or discordant selective pressures on gene regions among populations. While suggestive, correlational studies are unable to rigorously examine two populations for selective forces that differ at the level of an individual site, or a very short sequence region. Our test is capable of directly testing for such differences, including the case when we are only concerned with a subset of tree branches (e.g., internal or tips), and provides a rigorous significance level for such comparisons. Recent studies [40] provide strong evidence that site-to-site variation in synonymous substitution is pervasive in many genes, especially in HIV. Furthermore, it has been shown that failure to model such rate variation can result in uncontrollable rates of false positives and misidentify variable sites under relaxed selective constraints as those under strong positive selection pressure [22]. We have demonstrated that the new tests yield well-controlled false positive rates and high (>95%) PPV on data simulated with parameters realistic for HIV evolution. Additionally, the methods have been implemented as a part of parallelized software package HyPhy [57], can be run very quickly (≈10 min per 74 sequence sample) on a small computer cluster, and lend themselves to practical investigation of statistical properties of the method based on simulations, which can be tailored to the specific dataset being analyzed.

Adaptation to the host occurs at many levels in HIV: to the intracellular, intra-individual, and intrapopulation levels we have added an interpopulation level. Novel statistical methodology has allowed us to discriminate adaptation occurring at the last two levels and to answer questions raised by earlier correlation studies [15,50]. We have shown that within-host adaptation is often transient and that the codons at which persistent substitutions occur (which would include the immunological footprint) are subject to a substantially stronger ongoing selective force than those at which transient substitutions are seen. The ability to distinguish transient from persistent substitutions could be important for the development of an effective vaccine [58], as well as opening new routes to the analysis of selection in other settings.

## Materials and Methods

### Phylogeny reconstruction and substitution models.

We used an iterative process [24] to reconstruct a phylogeny of the sample and to select an appropriate nucleotide substitution model, a special case of the general time-reversible Markov model [59]. Independent substitution bias parameters and branch lengths were fitted to each alignment, using the pruning algorithm [60], modified [61] for faster evaluation of phylogenetic likelihood functions, and numerical optimization routines, implemented in HyPhy [57], to obtain maximum likelihood parameter estimates (MLEs). The *MG*94 × *REV* codon model, which estimates synonymous 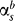 and nonsynonymous 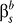 rates independently at every site of the alignment (and possibly differing between branches), was then fitted, while holding branch length and nucleotide substitution bias parameters fixed at MLE values obtained with a nucleotide model using the entire alignment. The rate matrix for this model is a modification of the *MG*94 [62] model (see also [40]), to allow for variable rates across different branches in the tree and to correct for all possible nucleotide substitution biases, given by

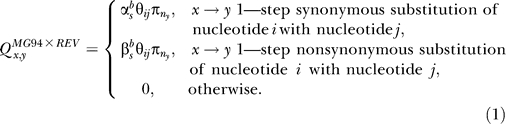



 To ensure time-reversibility we set *θ_ji_ = θ_ij_*. Because only the products of rates and times are estimable, one of the parameters *θ_ij_* cannot be identified, and we choose to set *θ_AG_ =* 1; *θ_ij_* estimates are obtained from the entire alignment and reflect the rate of substituting nucleotide *i* with nucleotide *j* relative to the A↔G substitution. π*_n_y__* denotes the relative frequency of the nucleotide in position *n* (1,2,3) in codon *y*. For instance, the target nucleotide for the synonymous ACG to ACT substitution is T in the third codon position, and its corresponding rate is 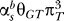 . Under *MG*94 × *REV,* the stationary frequency of codon *y* composed of nucleotides *i, j,* and *k* is the product of the constituent nucleotide frequencies, scaled to account for the stop codons. For sequences using the universal genetic code, these frequencies are given by


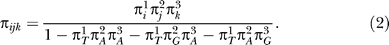


 Other genetic codes can be easily accommodated by adjusting the list of stop codons. The (*x*,*y*) entry of the transition probability matrix *T*(*t*) = exp(*Qt*) defines the probability of substituting codon *x* with codon *y* in time t ≥ 0. All data analyses were conducted on a 40-processor Linux cluster and all simulation studies were run on 64 processors of the Swansea Blue-C IBM cluster, using the message passing interface (MPI) distributed framework.

### Testing for temporal differences in evolution within a sample.

Every codon *s* can be endowed with a single synonymous rate *α_s_* and two nonsynonymous rates: 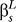 (for terminal branches, or leaves) and 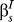 (for internal branches). If the latter two rates differ significantly, we deduce that evolution along internal branches (historical, e.g., influenced primarily by selection for transmission in HIV) and along terminal branches (recent, e.g., influenced by within-patient evolution in HIV) are subject to differing selective constraints. Formally,

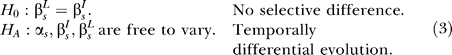
 A straightforward modification of the null hypothesis can be used to test for non-neutral evolution only along internal branches of the tree:

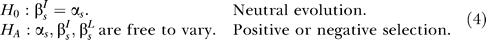



 We refer to the latter test as IFEL (internal fixed effects likelihood). Significance is assessed by the likelihood ratio test with one degree of freedom. Our simulations (see simulation strategy details below) have shown that the use of the 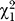 asymptotic distribution leads to a conservative test, and actual false positive rates (in our simulation scenario) are lower than the nominal significance level of the test (Figure S7). For a given sample size, the power of the test depends on the divergence level and the disparity between levels of selection between internal and terminal branches. For example, at *p* = 0.05, the overall power of the test to detect non-neutral evolution is only 25%. This rather low number can be partially explained by a large proportion of codon sites with low degree of polymorphism. Such sites are nearly impossible to classify within the current phylogenetic framework. However, if we narrow our focus to strongly selected sites (i.e., sites where 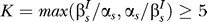 ) with an above average level of divergence (*α_s_* > 1), the power increases to 41%. For very strongly selected sites (K ≥ 16), the power is boosted to 68%. Overall, the PPV of the test is 98.8%.

### Population level adaptation test.

Given two partitions (Tips only *dN* > 0, or A for brevity, and Internal *dN* > 0, or B) of sites in an alignment, we performed a maximum likelihood fitting of the *MG*94 × *REV* model with each of the partitions having a single synonymous (*α_A_*,*α_B_*) and two nonsynonymous substitution rate parameters: the rate for internal branches (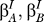 ), and that for terminal branches (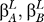 ). Rate parameters are shared by all codons in the partition and estimated by maximum likelihood, whilst branch length parameters are held at values estimated from the entire alignment previously. We then tested whether the average selective pressure, measured by the ratio *β^L^*/*α,* along terminal branches was different between partitions A and B. Formally,

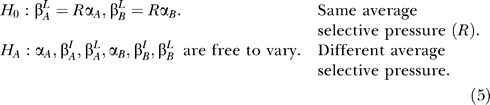
Significance was assessed by the likelihood ratio test with one degree of freedom using the asymptotic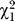 distribution of the LR statistic. Note that this test is an extension of the fixed sites approach of Yang and Swanson [63] to allow for variable selective pressures in different parts of the tree across partitions. When the sample size is small, the asymptotic distribution of the LR statistic may not be appropriate, hence we verified significance of the test using parametric bootstrap with 100 replicates.

### Testing for differential evolution between populations.

Having fitted 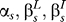 to each codon independently in sequences sampled from two different populations, we can test whether the selective pressure along internal branches was discordant between populations. We say that differential historical evolution has acted on codon *s* when 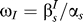 differs significantly between two populations. Formally,

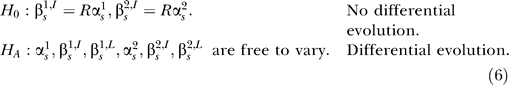
Significance of the difference can be assessed assuming the 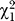 distribution for the likelihood ratio test statistic. Our simulations (see below) suggest that the 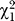 -based determination of significance leads to a conservative test (Figure S8) and actual false positive rates (in our simulation scenario) are lower than the nominal significance level of the test. For a given sample size, the power of the test depends on the divergence level and the disparity between levels of internal branch selection between the samples. The proportion of sites correctly identified as evolving under temporally differential selection also depends on how different the ratio 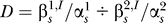 is from one. For example, at nominal *p* = 0.05, the overall power of the test to identify differential evolution along internal tree branches is merely 8%. Low overall power is attributable to the small extent of polymorphism at many codon positions and small sample sizes. However, for the sites with medium to high levels of divergence (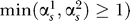 and where max(*D*,1/*D*)≥8, the power increases to 40%. If max(*D*,1/*D*)≥32, the power goes up to 64%. Overall, the PPV of the test is 98.2%.

### Multiple test correction.

Likelihood ratio tests for selection at an individual site have been applied in similar contexts in at least three studies [22,64,65]. If the main objective is to test whether or not there is evidence for selection *somewhere* in the sequence, based on the results of a series of site-by-site tests, then one would have to employ a multiple-test correction procedure—for example, the Bonferroni correction or a less conservative false discovery rates [66] approach. However, at the level of any given site, as argued in the three cited manuscripts, it is appropriate to use uncorrected *p*-values. Furthermore, our Type I error simulation studies (Figures S7 and S8) show that the size of the test at the level of an individual site is actually less than the nominal *p*-value.

### Simulation strategy.

Error rates and the power of the tests reported in the previous sections were derived using sequence data simulated under the following protocol. We have used trees, base frequencies, branch lengths (assuming neutral evolution), and nucleotide substitution biases fitted to the two ZA RT sample from our study, with 74 sequences each to simulate 100 (200 codons in each). A neighbor joining tree (using the Tamura–Nei distance metric [67]) was reconstructed from each data replicate and used for further inference, allowing us to investigate whether the power and error rates of the tests were unduly influenced by errors in phylogenetic reconstruction. Previous studies [22] and simulations results presented here (Figure S7) suggest that fixed effect likelihood methods are able to infer site-specific substitution rates accurately, on average, with moderate smoothing effects for larger rates (due to a fairly small sample size). With that in mind, we set out to generate sequences under a distribution of substitution rates that is similar to those which have influenced our real samples. Having fitted the IFEL model (and thus three rates: *α_s_,* 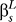 *,* and 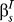 ) to all four samples, we pooled each type of estimated rates into the following seven bins: [0,0.25), [0.25,0.5), [0.5,1), [1,1.5), [1.5,2.0), [2.0,4.0), and [4.0,∞), and represented each bin with its midpoint (except the final bin, which was represented by 8). For each codon, we drew *α_s_*, 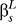 *,* and 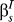 from the appropriate estimated rate distribution (also shown in Figure S8). Sampling from distributions with identical supports ensured that a sufficient proportion of sites was generated under the null distribution (e.g., 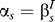 for IFEL and 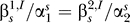 ). For the evaluation of the differential selection test, we picked successive pairs of simulations (1−2, 2−3, 3−4, … , 99−100) for a total of 99 runs of the analysis.

### Implementation.

All the tests have been implemented as scripts in the HyPhy [57] batch language and are either a part of the standard distribution of the package, or can be obtained upon request from the authors.

## Supporting Information

Figure S1 Joint PR and RT Phylogenies Inferred from Combined Sample Data Ethiopian and South African sequence samples appear reciprocally monophyletic, both in the maximum likelihood and in the 50% consensus Bayesian trees.
(112 KB EPS)

Figure S2 Inferred Evolutionary History at Codon 82 in RT The trees were rooted using subtype B reference sequences from the Los Alamos HIV database, and ancestral states were inferred using maximum likelihood under the MG94×012232 Dual GDD 3 × 3 [40] model of codon evolution.
(127 KB EPS)

Figure S3 Inferred Evolutionary History at Codon 98 in RT The trees were rooted using subtype B reference sequences from the Los Alamos HIV database, and ancestral states were inferred using maximum likelihood under the MG94×012232 Dual GDD 3 × 3 [40] model of codon evolution.
(134 KB EPS)

Figure S4 Inferred Evolutionary History at Codon 165 in RT The trees were rooted using subtype B reference sequences from the Los Alamos HIV database, and ancestral states were inferred using maximum likelihood under the MG94×012232 Dual GDD 3 × 3 [40] model of codon evolution.
(128 KB EPS)

Figure S5 Inferred Evolutionary History at Codon 177 in RT The trees were rooted using subtype B reference sequences from the Los Alamos HIV database, and ancestral states were inferred using maximum likelihood under the MG94×012232 Dual GDD 3 × 3 [40] model of codon evolution.
(136 KB EPS)

Figure S6 Inferred Evolutionary History at Codon 202 in RT The trees were rooted using subtype B reference sequences from the Los Alamos HIV database, and ancestral states were inferred using maximum likelihood under the MG94×012232 Dual GDD 3 × 3 [40] model of codon evolution.
(128 KB EPS)

Figure S7The Distribution of Rates Used to Simulate the Data, together with the Boxplots of Generating Rates and Corresponding Rates for Each of the Three Types of Rates at a SiteThe bottom right panel depicts the rate of false positives (identifying sites with 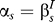 as non-neutrally evolving along internal branches) versus the significance level of the IFEL selection test. Solid gray line shows the expected error rate. Because the actual rate of false positives (for this simulation scenario) is lower than predicted by the significance level of the test, we deduce that the IFEL test behaves conservatively. (More detail is available in the Materials and Methods section.)
(487 KB EPS)

Figure S8False Positive Rate for the Differential Selection TestThe bottom right panel depicts the rate of false positives (identifying sites with 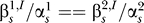 as evolving differentially along internal branches) versus the significance level of the differential selection test.
The solid gray line shows the expected error rate. Because the actual rate of false positives (for this simulation scenario) is lower than predicted by the significance level of the test, we deduce that the test behaves conservatively. (124 KB EPS)

## Abbreviations

IFEL: internal fixed effects likelihood
MHC: major histocompatability complex
MLE: maximum likelihood (parameter) estimate
PPV: positive predictive value
PR: protease transcriptase
RT: reverse transcriptase
